# Modeling Membrane Curvature Generation due to Membrane–Protein Interactions

**DOI:** 10.3390/biom8040120

**Published:** 2018-10-23

**Authors:** Haleh Alimohamadi, Padmini Rangamani

**Affiliations:** Department of Mechanical and Aerospace Engineering, University of California, San Diego, CA 92093, USA; halimoha@eng.ucsd.edu

**Keywords:** plasma membrane, spontaneous curvature, Helfrich energy, area difference elastic model, protein crowding, deviatoric curvature, hydrophobic mismatch

## Abstract

To alter and adjust the shape of the plasma membrane, cells harness various mechanisms of curvature generation. Many of these curvature generation mechanisms rely on the interactions between peripheral membrane proteins, integral membrane proteins, and lipids in the bilayer membrane. Mathematical and computational modeling of membrane curvature generation has provided great insights into the physics underlying these processes. However, one of the challenges in modeling these processes is identifying the suitable constitutive relationships that describe the membrane free energy including protein distribution and curvature generation capability. Here, we review some of the commonly used continuum elastic membrane models that have been developed for this purpose and discuss their applications. Finally, we address some fundamental challenges that future theoretical methods need to overcome to push the boundaries of current model applications.

## 1. Introduction

The ability of cellular membranes to bend and adapt their configurations is critical for a variety of cellular functions including membrane trafficking processes [1,2], fission [3,4], fusion [5,6], differentiation [7], cell motility [8,9], and signal transduction [10,11,12]. Defects or disruptions in these processes can lead to drawbacks in development and disease [13,14]. For example, changes in the level of cytosolic phospholipase $A_{2}$ (${\mathrm{cPLA}}_{2}\alpha$) enzyme affect the formation of transport vesicles from the Golgi to the plasma membrane [15]. This malfunction can cause diseases such as asthma [16], arthritis [17], cerebral ischemia [18], heart disease [19], and cancers [20]. Another example is remodeling tubular membranes in centronuclear myopathies (CNM) patients due to mutations in myotubularin (MTM1), amphiphysin2 (BIN1), or dynamin2 (DNM2) proteins [21]. Many of these protein families are associated with lipid homeostasis and membrane curvature generation.

The degree of the membrane deformability depends on lipid packing, which can affect membrane tension and the flow and diffusion of lipids in the plane of the membrane [22,23,24]. To dynamically reshape the membrane, cells rely on a variety of molecular mechanisms, ranging from forces exerted by the cytoskeleton [25,26,27] to the spontaneous curvature induced by the membrane–protein interactions [22,28,29,30]. Each mechanism generates unique surface stresses on the membrane and these surface stresses can be mapped onto the shape to understand the mechanical aspects of the membrane deformation [31,32,33,34]. The interplay between cellular membrane and membrane proteins is one of the major sources of the curvature production in cells. Membrane–protein interactions result not only from proteins that are integral to the membrane, but also from proteins that can bind to the membrane surface locally in response to signaling events such as scaffolding molecules or GTPases [28,29,35,36,37,38].

Many different mechanisms have been proposed for how proteins can generate curvature of the membrane. For the purposes of theoretical modeling and capturing the key physical principles, the broadly accepted mechanisms can be grouped into two main categories: (i) the hydrophobic insertion mechanism; and (ii) coat proteins with hydrophilic domains [29,39,40]. In the hydrophobic insertion mechanism, partially embedded amphipathic helices of the protein domains change the relative area of the two membrane leaflets. This area mismatch produces stresses, which result in membrane bending [41,42]. In contrast, when proteins are thought to coat the membrane, there is no insertion into the lipid bilayer and proteins simply oligomerize along the membrane surface [43,44]. In this case, it has been suggested that the steric pressure generated due to protein crowding and scaffolding drive the membrane deformation [45,46,47].

**Figure 1:**
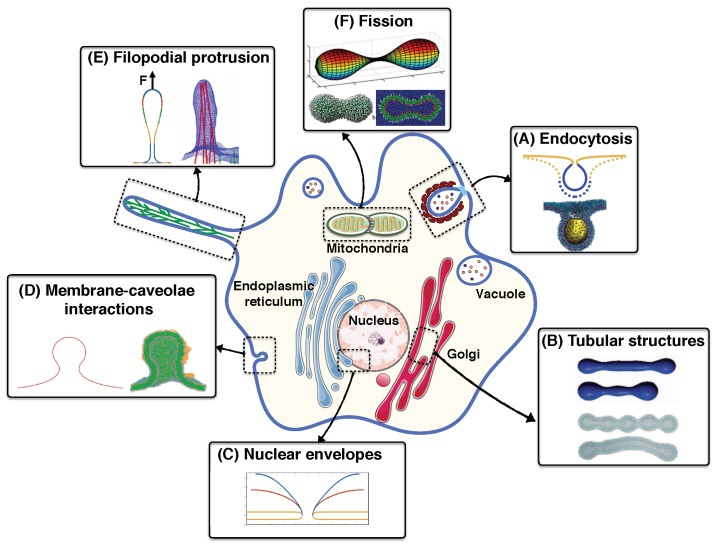
Membrane curvature generation in cells and associated modeling results: (**A**) Membrane budding in endocytosis. Reprinted with permission from references [66,67]. Copyright 2017 PNAS and Copyright 2018 PCCP; (**B**) Formation and stabilization of tubular membrane structures in the Golgi. Reprinted with permission from references [68,69]. Copyright 2013 PloS one and Copyright 2017 ACS Nano; (**C**) Change in the topology of nuclear envelopes. Reprinted with permission from reference [70]. Copyright 2016 PNAS; (**D**) Membrane invagination in caveolae. Reprinted with permission from references [71,72]. Copyright 2013 Soft matter and Copyright 2011 J. Phys. Chem. B.; (**E**) Actin force driven filopodia protrusion. Reprinted with permission from references [73,74]. Copyright 2015 PNAS and Copyright 2016 PLoS Comput Biol.; and (**F**) Mitochondrial fission. Reprinted with permission from references [75,76]. Copyright 2017 Front Physiol and Copyright 2007 J. Phys. Chem. B.

There are various methods to visualize membrane curvatures in situ or in reconstituted systems such as X-ray crystallography [48,49], nuclear magnetic resonance spectroscopy (NMR) [50,51], fluorescence microscopy [52,53], and electron microscopy (EM) [54,55]. Use of these techniques provides an opportunity for scientists to decipher vast amounts of information about the molecular machinery underlying the membrane shape transformations at high resolution. However, taking high resolution images is expensive and biological systems are very dynamic, making it challenging to experimentally quantify the role of a specific component, e.g., membrane–protein interactions, in biological phenomena [56,57,58]. The use of theoretical and computational approaches have become popular as complementary techniques to explore the mechanochemical aspects of membrane curvature generating mechanisms [59,60,61,62,63,64,65]. In Figure 1, some results from theoretical simulations of membrane deformation in endocytosis [66,67], tubular structures [68,69], nuclear envelopes [70], caveolae [71,72], filopodial protrusion [73,74], and fission [75,76] are represented.

In this review article, we mainly focus on the continuum models that incorporate the effects of membrane–protein interactions into the cell membrane curvature. In Section 2, we briefly introduce the basic components of biological membranes. Next, in Section 3, we outline different type of proteins and their importance in cellular processes by adjusting the membrane curvature. In Section 4, we present two different computational approaches for modeling membrane–protein interaction—molecular dynamics versus continuum models. In Section 5, we provide an overview of some of the popular continuum models for describing the constitutive relationships of the plasma membranes in contact with proteins. Finally, we conclude this review with a discussion on the challenges and possible future directions of the theoretical methods in Section 6.

## 2. Composition of Biological Membranes

**Figure 2:**
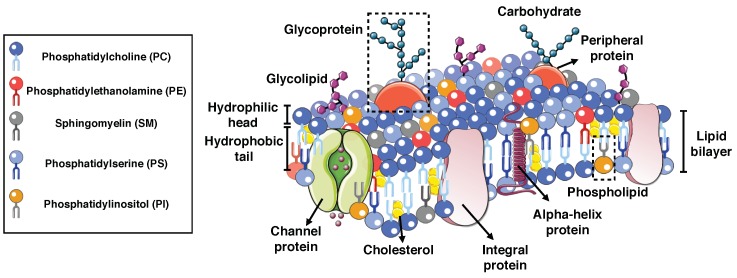
Schematic depiction of a cellular membrane highlighting its composition. There are two layers of amphipathic lipid molecules that self-assemble to form the bilayer. In each layer, the hydrophilic head groups form the outer surface and the hydrophobic tails face toward each other in the interior region. The distribution and organization of lipids and different proteins can vary from cell to cell. The cell membrane is composed of many different molecules including peripheral proteins, integral proteins, and carbohydrate molecules.

Biological membranes (BMs) form the outer boundary of living cells and compartments inside the cell. The main component of all biological membranes is a lipid bilayer, with a thickness of about 5–10 nm (see Figure 2) [77,78,79]. Proteins are the second major component of cell membranes in which the weight ratio of the lipids to membrane proteins can vary from 20% to 70%, depending on the cell type [78,80,81]. Proteins in cell membranes are classified into two categories: integral and peripheral proteins [82,83] (see Figure 2). The third major component of BMs is carbohydrate molecules, which are found on the extracellular sides of cell membranes [84,85]. We briefly survey the two different classes of membrane proteins (integral and peripheral proteins), their functions, and their structures in cell membranes in what follows.

### 2.1. Integral Proteins

Integral proteins are embedded permanently in the membrane by hydrophobic and electrostatic interactions [86,87]. Therefore, removing integral proteins from lipid bilayer is only possible by the use of detergents or nonpolar solvents that break down the strong membrane–protein interactions. The most common type of integral proteins are transmembrane proteins, which span across the lipid bilayer such that one end contacts the cell interior and the other end touches the exterior. Many of the integral membrane proteins function as ion channels or transporters. In addition, cell surface receptors, linkers, and enzymatic proteins are all classes of integral membrane proteins [88].

### 2.2. Peripheral Proteins

Peripheral proteins more or less temporarily bind to the surface of the membrane with weak interactions [86,89]. This means that unlike integral proteins, peripheral proteins can be separated from the lipid bilayer by either altering the pH or the salt concentration of the cell culture medium [78]. The primary role of peripheral proteins is to provide a point of attachment for other components to the cell membrane. For instance, both membrane cytoskeleton and components of the extracellular matrix are linked to the cell membrane through peripheral proteins. This helps the cell maintain its shape while the membrane remains flexible to bend as needed for various cellular functions [90]. Besides the structural supports, peripheral proteins are involved in many other functions including cell–cell communication, energy transduction, and molecule transfer across the membrane [90].

## 3. Membrane Curvature Generation due to Proteins

### 3.1. Conical and Inverted Conical-Shaped Proteins

The shape of transmembrane proteins can be approximated as conical or inverted conical shapes [91,92,93]. These proteins are thought to insert into the membrane, distort the packing of the lipids, and thus impose local negative or positive curvature to the underlying membrane [94]. Cellular membranes can bend either towards or away from the cytoplasm. The membrane curvature is considered positive if the membrane curves toward the cytoplasm, and the curvature is negative if the membrane curves away from the cytoplasm [22,23]. The attached conical or inverted conical-shaped proteins induce membrane bending due to insertion causing a wedge effect, which can possibly be associated and amplified by oligomerization, protein crowding, or hydrophobic mismatch [95]. In addition to the direct effects of conical or inverted conical-shaped proteins, the membrane-mediated repulsive interactions between two embedded proteins result in a change in the membrane curvature [96,97]. Two classical examples of conical transmembrane proteins are potassium ion channels and Nicotinic acetylcholine receptors, which can generate long-range membrane deformations [98].

### 3.2. BIN-Amphiphysin-Rvs Domain Proteins

BIN-Amphiphysin-Rvs (BAR) domain proteins are banana-shaped proteins that can both sense and influence membrane curvature [99,100]. BAR domain proteins are made of three coiled core helices attached to multiple positively charged residues [28,101]. Endophilin, Arfaptin, Amphiphysin, Syndapins, Nadrin, and Oligophrenin are all membrane of the large family of BAR domain proteins [102]. BAR domains are categorized in three groups based on the structure [101]: a classical BAR domain (including N-terminal amphipathic helix BAR (N-BAR) domain family proteins),

extended Fes-CIP4 homology BAR (F-BAR), and IRSp53-MIM homology domain (IMD)/Inverse BAR domain (I-BAR). BAR proteins are known to induce membrane curvature by two mechanisms: scaffolding (imposing their intrinsic shapes on the membrane substrate) [28] and insertion of amphipathic helices at the interface of the lipid bilayer, locally creating a wedge effect [103]. In terms of functionality, BAR domain proteins are involved in numerous cellular processes including endocytosis, exocytosis, apoptosis, and cell–cell fusion [101]. For example, in the formation of a filopodial protrusion, the driving force of the actin polymerization enhances by membrane bending due to BAR domain scaffolding [104]. Indeed, the effect of BAR domain proteins scaffolding on the filopodia formation can play a central role in cancer invasion and the formation of an invadopodia [105,106].

### 3.3. Coat Proteins

To regulate some cellular trafficking phenomena, multiple proteins need to bind to the membrane and form a coat complex such as clathrin, coat protein complex I (COPI), and COPII [107]. These protein assemblies can act as a scaffold to impose their spherical curvature to the underlying membrane [28]. However, other components of the coat can contribute to the membrane bending through helix insertion into the bilayer or adaptor-protein crowding [28]. Clathrin-mediated endocytosis (CME), coated COPI transport vesicles between the endoplasmic reticulum (ER) and the Golgi, and endosomal sorting complexes required for transport (ESCRT) protein assemblies at the neck of endocytic buds are all examples of membrane remodeling due to the activity of the coat proteins [108,109,110].

## 4. Theoretical Models of Biological Membranes

### 4.1. Mechanical Viewpoint

Theoretical approaches are complementary techniques that have been developed in the last few decades to understand how cells regulate their function through geometry, mechanics, and signaling [11,58,111,112,113]. In general, theoretical approaches can be classified into discrete and continuum models. In discrete models, the equations of the atoms’ motion in interaction with each other are solved by Molecular Dynamics (MD) or Coarse-Grained (CG) simulation techniques [114,115]. Tracing all atoms in a system makes this model suitable for exploring the nature of biological processes at the molecular level that are typically very difficult to detect experimentally such as the biochemistry underlying the lipid–lipid or lipid–protein interactions. However, the high computational cost of MD or CG simulations limit the applications of discrete models to phenomena at nanoscopic length and time scales [111,116,117].

On the other hand, the continuum approach treats the membrane as a continuous surface with average properties [111]. Indeed, the small length scale of the membrane constituents (∼3–6 nm) compared to the length scales of the biological phenomena (∼100 nm-μm), allows us to define the complex membrane as a single continuum surface [111]. The most popular and widely used model in continuum framework is the Helfrich model, which was proposed in 1973 [118]. In this model, the membrane is considered as a thin elastic shell that can bend such that at all times the lipids remain aligned and normal to the membrane surface. In addition, this model presumes that the curvature of the membrane is much larger than the thickness of the bilayer [118]. Under these assumptions, Helfrich proposed an energy function for the system that depends only on the mean and Gaussian curvatures of the membrane as [118]

(1) $$W_{\mathrm{Bending}}=\int_{\omega }\left(2\kappa H^{2}+\kappa_{G}K\right)dA,$$

where *W* is total strain energy of the membrane due to bending, *H* is the membrane mean curvature, *K* is the membrane Gaussian curvature, and κ and $\kappa_{G}$ are membrane properties which are called the bending and Gaussian moduli, respectively. The integration in Equation (1) is over the entire membrane surface area ω and dA is a differential area element. We describe the geometrical concepts of curvature of manifolds in Box A.

### 4.2. Simulation Techniques

From a mechanical perspective, cell membrane deformation can be characterized by balance laws for mass and momentum. Simplifying these mass and momentum conservation equations in a continuum framework results in a set of partial differential equations (PDEs) [119]. To solve the PDEs, we first need to define the constitutive relationship for the membrane deformation, for example, the Helfrich bending energy (Equation (1)). Other forms of suggested constitutive equations including the effects of proteins are presented in Section 5.

Besides the need for a constitutive equation, the derived PDEs from cell mechanics are usually higher order and highly nonlinear differential equations. Therefore, in most cases, analytical solutions are not possible and the equations are often solved numerically. Over the last few decades, various computational approaches have been developed to solve the set of governing PDEs including the boundary value problem for axisymmetric coordinates [32,66,73,120,121], different finite element methods [122,123,124], Monte Carlo methods [125,126,127], finite difference methods [128,129], and the phase field representation of the surface [130,131,132]. Each of these methods has its own advantages and disadvantages and, depending on the complexity of the problem, one or more of them can be implemented.

A major challenge in modeling membrane–protein interactions is identifying a constitutive relationship that captures the different levels of complexities associated with membrane–protein interactions. In what follows, we discuss some of the popular models used for such purposes along with their applications. We then discuss where new constitutive relationships are needed and how these can be experimentally parameterized.

## 5. Continuum Elastic Energy Models of Membrane–Protein Interactions

### 5.1. Spontaneous Curvature Model

**Figure 3:**
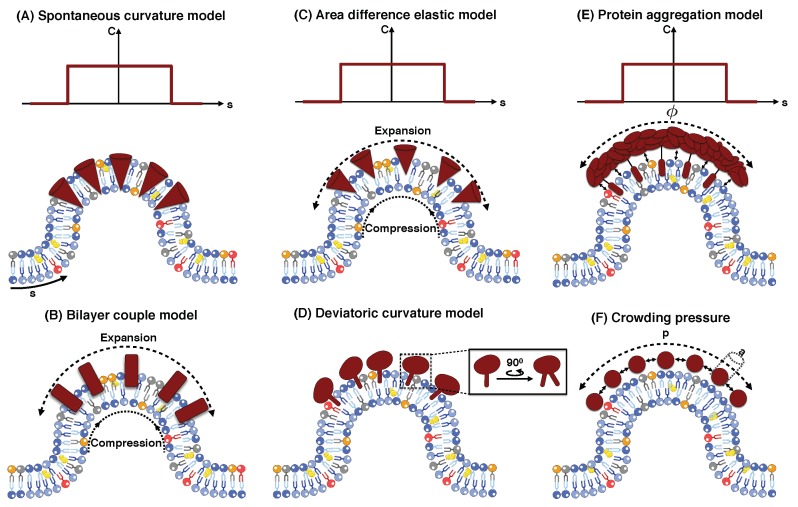
Cartoon models of the mechanisms of membrane curvature generation due to protein (shown in red) interactions in different continuum elastic models. (**A**) Local protein interactions with membrane produce a spontaneous curvature field. s is the arc length parameterization along the membrane and C is the induced spontaneous curvature. (**B**) The asymmetric insertion of conical proteins on one side of the membrane results in the expansion of the upper leaflet and compression of the lower leaflet. (**C**) Asymmetric insertion of proteins into the lipid bilayer induces both local spontaneous curvature and surface stresses due to membrane leaflets expansion/compression. (**D**) Rotationally non-symmetric proteins generate anisotropic curvature. (**E**) Aggregated proteins on the membrane surface create a spontaneous curvature field and also have entropic interactions with the membrane. Here, ϕ represents the relative density of the accumulated proteins. (**F**) The induced pressure (p) by crowding proteins drives membrane bending. a is the surface area occupied by one protein.

In the spontaneous curvature (SC) model, it has been suggested that the interaction between proteins and surrounding lipids changes the local membrane properties, particularly the preferred or spontaneous curvature of the membrane [29,133,134,135]. In this case, the induced spontaneous curvature is a parameter that reflects a possible asymmetry between the two leaflets of the bilayer. This can be the result of any membrane bending mechanisms such as phase separation of membrane proteins into distinct domains, amphipathic helix or conically-shaped transmembrane protein insertion, protein scaffolding, or protein crowding (Figure 3A). In reality, a combination of all these mechanisms can occur simultaneously; as a result the local value of spontaneous curvature can then be interpreted as a single measure of the curvature-generating capability of the membrane–protein interaction [28,29]. In a continuum framework, the most common model for induced spontaneous curvature is the modified version of Helfrich energy (Equation (1)), given in [73,134,136,137].

Box A **Curvatures of surfaces.**

**Figure A1:**
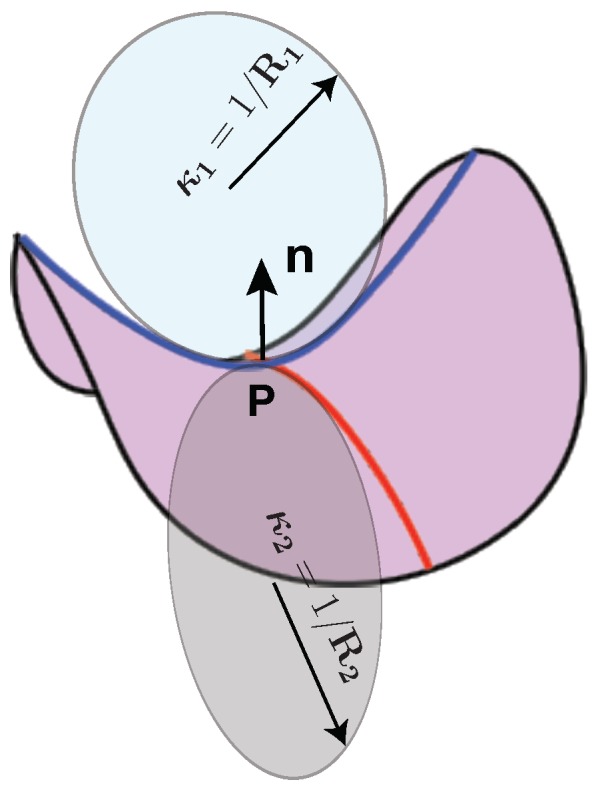
Principal curvatures of a surface.

Let us consider the membrane as a two dimensional surface in a three-dimensional Euclidean space (Figure A1). At each point on the surface, there are two curvatures, $\kappa_{1}$ and $\kappa_{2}$, which characterize the shape of the surface [138,139]. These two curvatures are called principal curvatures and by the definition their values are the reciprocal of the radius of the osculating circle at the point (**P**) ($\kappa_{1}=1/R_{1}$ and $\kappa_{2}=1/R_{2}$ in Figure A1) [138,139]. The values of these curvatures can be positive or negative. The curvature is positive if the curve turns in the same direction as the normal vector to the surface (n), otherwise, it is negative [138,139]. The average the product of two principal curvatures give the mean (*H*) and the Gaussian (*K*) curvatures, respectively, as [138,139]

(A.1) $$H=\frac{\kappa_{1}+\kappa_{2}}{2}\;\mathrm{and}\;K=\kappa_{1}\kappa_{2}.$$

**Figure A2:**
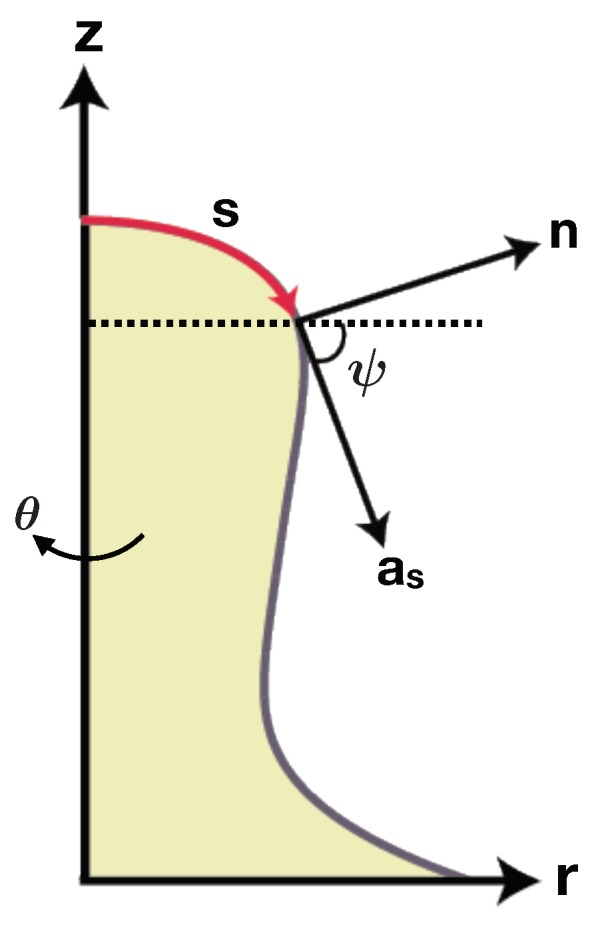
Axisymmetric coordinates with z as the axis of rotation.

For a rotationally symmetric surface, as shown in Figure A2, we can define the position of each point on the surface as a function of arc length (*s*) such as

(A.2) $$r\left(s\right)=r\left(s\right)e_{r}\left(\theta \right)+z\left(s\right)k,$$

where r(s) is the radius from axis of revolution, z(s) is the elevation from a base plane, and $\left(e_{r},e_{\theta },k\right)$ forms the coordinate basis. Since $r^{2}\left(s\right)+z^{2}\left(s\right)=1$, we can define the angle ψ such that (Figure A2)

(A.3) $$a_{s}=\cos\left(\psi \right)e_{r}+\sin\left(\psi \right)k\;\mathrm{and}\;n=-\sin\left(\psi \right)e_{r}+\cos\left(\psi \right)k,$$

where $a_{s}$ and n are the unit tangent and normal vectors to the surface, as shown in Figure A2. We now can define the two principal curvatures as

(A.4) $$\kappa_{1}=\psi^{\prime }\;\mathrm{and}\;\kappa_{2}=\frac{\sin(\psi )}{r},$$

where ${\left(\cdot \right)}^{\prime }=d\left(\cdot \right)/ds$ is the partial derivative with respect to the arc length. With the two principal curvatures, the curvature deviator (*D*) in anisotropic condition is given by

(A.5) $$D=\frac{1}{2}\left(\frac{\sin(\psi )}{r}-\psi^{\prime }\right).$$

(2) $$W_{\mathrm{SC}}=\int_{\omega }\left(2\kappa {\left(H-C\right)}^{2}+\kappa_{G}K\right)dA,$$

where *C* is the spontaneous curvature and its effective strength depends on the membrane composition, temperature, the membrane thickness, the protein density, and the membrane area coverage by proteins [118,140].

Modeling the net effect of membrane–protein interaction as an induced spontaneous curvature (Equation (2)) has provided great insight into various aspects of membrane deformation, from caveolae and endosomal sorting complexes to cylindrical shapes of membrane ER [141,142,143]. By using the SC model, recent studies have shown for example how a line tension at a lipid phase boundary could drive scission in yeast endocytosis [32,144,145], or how a snap-through transition from open U-shaped buds to closed buds in CME is regulated by the membrane tension [66,73]. Furthermore, the experimentally observed change in the membrane tension (spontaneous tension) in response to protein adsorption [146,147,148], can be explained in the context of the SC model [120,136,140]. The SC model has also been used to elucidate the role of varying membrane tension due to protein-induced spontaneous curvature [120,136,140]. While the SC model has been very effective in capturing large-scale deformations of the membrane, it does not take into account the protein density or the curvature induced by individual moieties.

### 5.2. Bilayer Couple Model

To go beyond an idealized single manifold description of a membrane, the bilayer couple (BC) model was proposed by Sheetz and Singer in 1974 [149]. The basic assumptions in this model are that each lipid molecule has a fixed area and there is no lipid exchange between the two leaflets of the bilayer. Thus, any asymmetrical protein insertions into the inner and outer surfaces of the membrane can cause an area mismatch between the two leaflets. This mismatch creates in-plane compression in one leaflet and extension in the other leaflet, resulting in the membrane deformation to release the induced stress (Figure 3B) [29,150]. For a thin lipid bilayer with thickness (*d*), the area difference between the leaflets (ΔA) can be expressed in terms of the mean curvature (*H*) as

(3) $$\Delta A=2d\int_{\omega }HdA.$$

Here, instead of having a spontaneous curvature term in energy, a “hard” constraint on the area difference between the leaflets (Equation (3)) regulates the membrane curvature. This difference in the mechanism of curvature generation of SC and BC models distinguishes their predictions for the same membrane deformation [150]. For example, in the case of membrane budding transition due to thermal expansion, the SC model predicts that the membrane budding is discontinuous, while the BC model predicts intermediate pear-shaped structures of the vesicle and that the transition of shapes is continuous [150].

### 5.3. Area Difference Elasticity Model

In 1980, the area difference elasticity (ADE) model was developed by Svetina et al. [151,152] to combine both SC and BC models including the missing macroscopic details of membrane bending phenomena. To better explain the physics underlying this model, we consider a flat membrane that bends downward due to different protein concentrations on two sides of the membrane (Figure 3C). This bending, based on the single sheet descriptions of the membrane in the SC model, gives rise to the spontaneous curvature term in the energy equation (Equation (2)). However, if we treat each leaflet as an independent elastic plate—as suggested in the BC model—we can then see that, besides the curvature, the area of each monolayer will also change. For example, in Figure 3C, the outer monolayer is stretched and the inner one is compressed. The energy associated with the membrane bending and this relative change in the monolayers areas is given by [150,153,154]

(4) $$W_{\mathrm{ADE}}=\underset{\mathrm{Bending}\;\mathrm{energy}}{\underset{︸}{\int_{\omega }\left(2\kappa {\left(H-C\right)}^{2}+\kappa_{G}K\right)dA}}+\underset{\mathrm{Elastic}\;\mathrm{stretching}\;\mathrm{energy}}{\underset{︸}{\frac{\kappa_{r}}{2Ad^{2}}{\left(\Delta A-\Delta A_{0}\right)}^{2}}},$$

where $\kappa_{r}$ is called the nonlocal membrane bending modulus and *A* is the total surface area of the neutral plane. $\Delta A_{0}$ and ΔA are the relaxed initial and bent area differences between the membrane leaflets. respectively ($\Delta A_{0}=A_{0,\mathrm{out}}-A_{0,\mathrm{in}}$ and $\Delta A=A_{\mathrm{out}}-A_{\mathrm{in}}$, in which $A_{\mathrm{out}}$ is the area of the outer layer and $A_{\mathrm{in}}$ is the area of the inner layer). In Equation (4), κ and $\kappa_{r}$ are both in order of $K_{a}d^{2}$, where $K_{a}$ is the area stretching modulus of the bilayer [150,154,155]. This means that. in any membrane deformation, both terms, the bending and the elastic stretching energies, are comparable and must be considered simultaneously. Using the ADE model, researchers for the first time could numerically simulate the shape transformations of the human red blood cell from stomatocyte to discocyte and to echinocyte [155,156,157,158]. In addition, by using the ADE model, the experimentally observed vesicle shapes were mapped onto a theoretical phase diagram, enabling theoreticians to predict the range of parameters in which the vesicles may become unstable [150,153]. These predictions have been very useful for detecting unstable shapes, which is challenging to do experimentally.

### 5.4. Deviatoric Curvature Model

In the SC model, the induced spontaneous curvature was assumed to be isotropic, meaning it has the same value in all directions (see Box A). However, not all proteins are rotationally symmetric and some can have intrinsically anisotropic curvatures such as banana-shaped BAR domain proteins (Figure 3D) [99,159,160]. These proteins can produce different curvatures in different directions, which is required for the formation of nonspherical structures such as membrane tubular protrusions [161,162]. To take into account the anisotropic contribution of protein coats or inclusions in the continuum approach, Kralj-Iglic et al. proposed a deviatoric elasticity (DE) model [163]. In this model, each complex protein structure is simplified as a one-dimensional curve that lies on the membrane. The orientation and the position of the proteins in the plane of the membrane are important factors since an additional term is needed to adjust the actual local curvature of the membrane to the intrinsic curvatures of the proteins [163,164]. The membrane free energy that was suggested by the DE model is given as [163,165]

(5) $$W_{\mathrm{DE}}=\int_{\omega }\underset{\mathrm{Bending}\;\mathrm{energy}}{\underset{︸}{(2\kappa {\left(H-C\right)}^{2}+\kappa_{G}K}}+\underset{\mathrm{Deviatoric}\;\mathrm{mismatch}}{\underset{︸}{2\kappa {\left(D-D_{0}\right)}^{2})}}dA,$$

where *D* is the membrane curvature deviator and $D_{0}$ is the spontaneous membrane curvature deviator. Since the DE model was proposed, there have been many modeling efforts to explain how the accumulation of BAR proteins in membrane necks stabilize membrane tubular protrusions without the support of the cytoskeleton [166,167,168,169]. Derivation of the Euler–Lagrange governing equations by a variational approach [170] provides a platform to systematically explore the impact of the induced stresses by anisotropic curvatures on the morphology of tubular structures [32].

### 5.5. Protein Aggregation Model

Aggregation of cytosolic proteins on the membrane surface or phase separation of bilayer proteins into specific domains have been observed in many biological processes [171,172,173,174]. This aggregation of proteins not only creates a concentration field on the membrane surface but also results in additional contributions to the membrane energy due to compositional heterogeneity and the entropic interactions of bulk proteins with the lipid bilayer (Figure 3E) [175,176,177]. While the exact form of the free energy is still a matter of debate and has not been verified experimentally, a simple model based on thermodynamic arguments is given as [175,176,178]

(6) $$\begin{array}{cc} W_{\mathrm{Aggregation}}= & \int_{\omega }\underset{\mathrm{Bending}\;\mathrm{energy}}{\underset{︸}{(2\kappa {\left(H-C\right)}^{2}+\kappa_{G}K}}+\underset{\mathrm{Entropic}\;\mathrm{energy}}{\underset{︸}{\frac{T}{a^{2}}\left(\phi \ln\phi +\left(1-\phi \right)\ln\left(1-\phi \right)\right)}}\\ & +\underset{\underset{\mathrm{aggregation}}{\mathrm{Energy}\;\mathrm{due}\;\mathrm{to}\;\mathrm{protein}}}{\underset{︸}{\frac{J}{2a^{2}}\phi \left(1-\phi \right)}}+\underset{\underset{\mathrm{heterogeneity}}{\underset{\mathrm{to}\;\mathrm{compositional}}{\mathrm{Energy}\;\mathrm{penalty}\;\mathrm{due}}}}{\underset{︸}{\frac{J}{4}{\left(\nabla \phi \right)}^{2})}}dA, \end{array}$$

where *T* is the environment temperature, *a* is the surface area occupied by one protein, ϕ is the relative density of the proteins, and *J* is the aggregation potential (J>0 represents attractive interactions and J<0 represents repulsive interactions). In Equation (6), the first term is the conventional Helfrich bending energy with induced spontaneous curvature [118]. The second term represents the entropic contribution due to the thermal motion of proteins in the membrane [175,179]. The third term gives the aggregation energy, and the last term describes the energetic penalty for the spatial membrane composition gradient [175,178,179]. This model was used to conduct theoretical analyses of dynamic phase transitions of coupled membrane–proteins–cytoskeleton systems in membrane protrusions such as microvilli and filopodia [175,180,181,182]. This model also reveals an interesting fact that, in addition to the induced deviatoric spontaneous curvature of the BAR domain proteins, the associated energy with their aggregation at membrane necks facilitates the stability of tubular structures [169,183].

The aggregation energy in Equation (6) is a representative of the direct protein–protein interactions in protein assemblies. However, there are indirect membrane-mediated interactions of proteins which result from the local changes in the membrane curvature, membrane structure, or membrane fluctuations [96,159,184,185]. For example, in the case of loose BAR domain assemblies, it is experimentally observed that the induced local membrane curvature due to protein binding generates a strong attractive interaction between two side-to-side crescent-shaped proteins without any direct protein–protein interactions [96,186]. This attraction is a key factor for the aggregation and cooperative action of BAR domain proteins during the formation of membrane tubular structures. Furthermore, coarse-grained simulations of membrane remodeling have shown that curvature-inducing proteins or particles can aggregate and bend the membrane even in the absence of direct attractive/repulsive interactions [111,187]. A major open question in the field is the relationship between protein density, size, and spontaneous curvature. Although current models use a linear proportionality [120,176,188], this choice of functions is critical in determining the energy.

### 5.6. Protein Crowding

The essence of the crowding mechanism is that the lateral collisions between the membrane-bound proteins on one side of the membrane generate a steric pressure that causes the membrane to bend away from the proteins (Figure 3F) [46,189,190]. As the density, the size, or the mobility of the bound proteins increases, the induced steric pressure becomes larger, which results in a more significant membrane bending [46,47]. Modeling the free energy associated with protein crowding is more difficult because it profoundly depends on the specific composition of the underlying membrane as well as the lateral confinement of the membrane-bound proteins [191,192]. However, in a recent paper, a simple 2D hard-sphere gas model based on the Carnahan–Starling approximation has been proposed to describe the free energy of the crowding mechanism [46,193]. To better visualize this, let us consider a membrane that is crowded with different protein concentration on each side as shown in Figure 3. If we model each protein as a hard-sphere gas particle that exerts a certain pressure on the membrane surface, the work that is done by this pressure to bend the membrane according to the standard thermodynamics is given by [194]

(7) $$W_{\mathrm{Crowding}}=\int p_{\mathrm{in}}dA_{\mathrm{in}}+\int p_{\mathrm{out}}dA_{\mathrm{out}},$$

where $p_{\mathrm{in}}$ and $p_{\mathrm{out}}$ are the induced steric pressure by the crowding proteins on the inner and the outer side of the membrane, respectively. This induced pressure (denoted by *p* here ) for a 2D hard-sphere gas protein can be expressed as [192,195,196]

(8) $$p=\frac{k_{B}T}{a}p_{R}\left(\phi \right),$$

where $k_{B}$ is the Boltzmann constant and $p_{R}\left(\phi \right)$ is the reduced gas pressure depending on the relative density of the protein as [196]

(9) $$p_{R}\left(\phi \right)=\phi \left(1+2\phi \frac{1-\frac{7}{16}\phi }{{\left(1-\phi \right)}^{2}}\right).$$

Equation (9) is known as a 2D version of the Carnahan–Starling equation. Protein crowding is a recently discovered curvature generating mechanism that has challenged some conventional paradigms about the role of molecular machinery in a robust cell shape change [45,46,47,197,198,199]. Stachowiak et al. reported that confining a sufficiently high concentration of his-tagged green fluorescent proteins (GFP) to a local region can deform the membrane into buds or tubules in the absence of any protein insertion into the lipid bilayer [46,197]. Later, Snead et al. showed that crowding among membrane-bound proteins can also drive membrane fission [45]. This paper predicts that the large disordered domains of BAR proteins induce crowding pressure that promotes membrane fission instead of stabilizing the membrane [200].

### 5.7. Hydrophobic Mismatch

Transmembrane proteins embedded in the cell membrane have hydrophobic regions that are in contact with hydrophobic regions (lipid acyl chain) of the lipid bilayer. The difference between the thicknesses of hydrophobic regions of a transmembrane protein ($d_{p}$) and the lipid bilayer ($d_{l}$) is called the hydrophobic mismatch. Energetically, it is favorable that both hydrophobic regions have approximately the same thickness to prevent the exposure of the hydrophobic surfaces to the hydrophilic environment. However, it is impossible to avoid a mismatch because there are various proteins with different lengths in a single membrane [201,202] and a single protein can be surrounded by lipid bilayers with different thicknesses [203,204].

**Figure 4:**
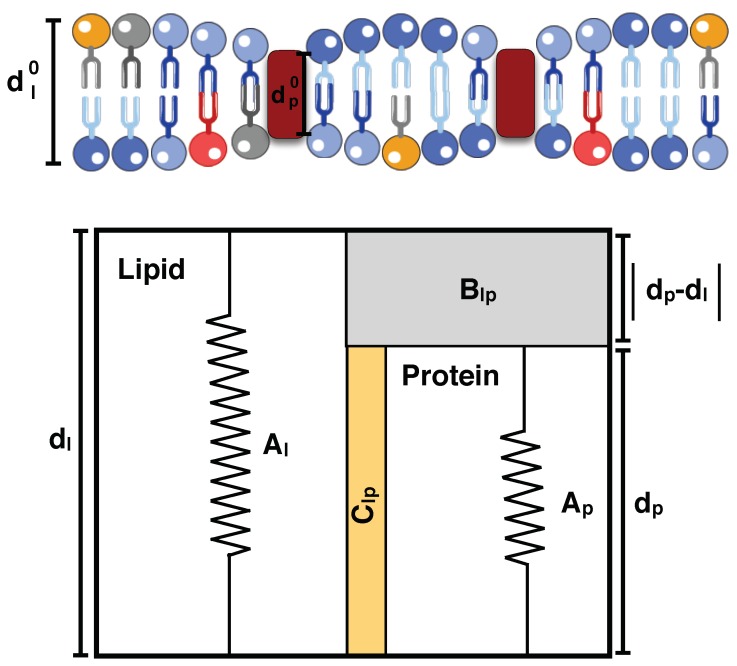
Mattress model representation of the hydrophobic mismatch phenomena [208]. Both protein and lipid bilayer are modeled as one-dimensional springs with constants $A_{p}$ and $A_{l}$, respectively. $d_{l}^{0}$ and $d_{p}^{0}$ are the initial lengths, and $d_{l}$ and $d_{p}$ are the final lengths of the lipid bilayer and the protein after deformation, respectively. The gray area corresponds to the hydrophilic region with the strength of $B_{lp}$, and the yellow region indicates the adhesive region with adhesive interactions strength of $C_{lp}$.

Several theoretical approaches have been developed to incorporate the energy cost and the thermodynamic effects of membrane–protein interactions in term of hydrophobic mismatch [205,206,207,208]. The mattress model is one of the most well-known models that was proposed by Mouritsen and Bloom in 1984 [208]. In this model, both protein and lipid bilayer (called a mattress) are characterized by one dimensional springs with constant $A_{p}$ and $A_{l}$, respectively [208] (Figure 4). There are three sources of energy in this model. First, elastic energy ($W_{\mathrm{Mattress}-\mathrm{Elastic}}$) due to the vertical deformation of the two springs relative to their individual equilibrium lengths ($d_{p}^{0}$ and $d_{l}^{0}$) given by [208]

(10) $$W_{\mathrm{Mattress}-\mathrm{Elastic}}=n_{l}A_{l}{\left(d_{l}-d_{l}^{0}\right)}^{2}+n_{p}A_{p}{\left(d_{p}-d_{p}^{0}\right)}^{2},$$

where $n_{l}$ and $n_{p}$ are the number of molecules in the lipid bilayer and protein domains, respectively. The second source of energy is due to the indirect lipid–protein interactions induced by the hydrophobic mismatch ($W_{\mathrm{Mattress}-\mathrm{hydrophobic}}$). Based on the standard regular solution theory, this hydrophobic energy is given by [209]

(11) $$W_{\mathrm{Mattress}-\mathrm{hydrophobic}}=\frac{n_{l}n_{p}}{n_{l}+n_{p}}B_{lp}\left|d_{p}-d_{l}\right|,$$

where $B_{lp}$ represents the strength of the hydrophobic interactions. The last source of energy is due to the direct protein–lipid interactions which has been modeled by an attractive adhesive interaction ($W_{\mathrm{Mattress}-\mathrm{adhesive}}$) as [208]

(12) $$W_{\mathrm{Mattress}-\mathrm{adhesive}}=\frac{n_{l}n_{p}}{n_{l}+n_{p}}C_{lp}min\left(d_{p},d_{l}\right),$$

where $C_{lp}<0$ shows the strength of the adhesive interactions between molecules. Therefore, the total energy associated with the mattress model is written as

(13) $$W_{\mathrm{Mattress}}=n_{l}A_{l}{\left(d_{l}-d_{l}^{0}\right)}^{2}+n_{p}A_{p}{\left(d_{p}-d_{p}^{0}\right)}^{2}+\frac{n_{l}n_{p}}{n_{l}+n_{p}}B_{lp}\left|d_{p}-d_{l}\right|+\frac{n_{l}n_{p}}{n_{l}+n_{p}}C_{lp}min\left(d_{p},d_{l}\right).$$

There are different adaptation mechanisms that either the protein or the bilayer can utilize to avoid the energy cost of the hydrophobic mismatch [203,210]. For example, for positive ($d_{l}<d_{p}$) or negative ($d_{l}>d_{p}$) mismatch, the lipid bilayer can be stretched or compressed, respectively, to adjust the length of hydrophobic regions [211,212]. Another possibility is when the hydrophobic part of a transmembrane protein is too thick or too short as compared to the hydrophobic bilayer thickness. In this case, protein aggregation on the membrane or protein surface localization can efficiently minimize the exposed hydrophobic area [213,214]. In addition, for proteins that have helices that are too long compared to the thickness of the membrane, helix tilt is one possible mechanism to reduce the protein effective hydrophobic length [203,215,216]. Effectively, the hydrophobic mismatch of integral membrane proteins is a clustering mechanism. However, this mechanism can generate membrane curvature depending on other membrane–protein interactions.

In addition to the models described above, there are additional considerations to the energy that have been suggested by numerous studies such as higher order bending terms [139,217,218], lipid volume constraints [219], the impact of protein shape on membrane deformation [220], and the electrostatic energy between a membrane and proteins [221,222,223,224].

## 6. Future Perspective and Challenges

Although the models discussed above have provided insight into the molecular machinery of cell shape regulation, all of them have been developed based on simplifying assumptions that need to be revisited in the pursuit of closing the gap between experiment and theory. To achieve this goal, multidisciplinary efforts among physicists, mathematicians, engineers, and biologists are required to match different pieces of this cell biology puzzle.

Here, we highlight some current challenges that we believe must be considered in the next generation of continuum models.

**Figure 5:**
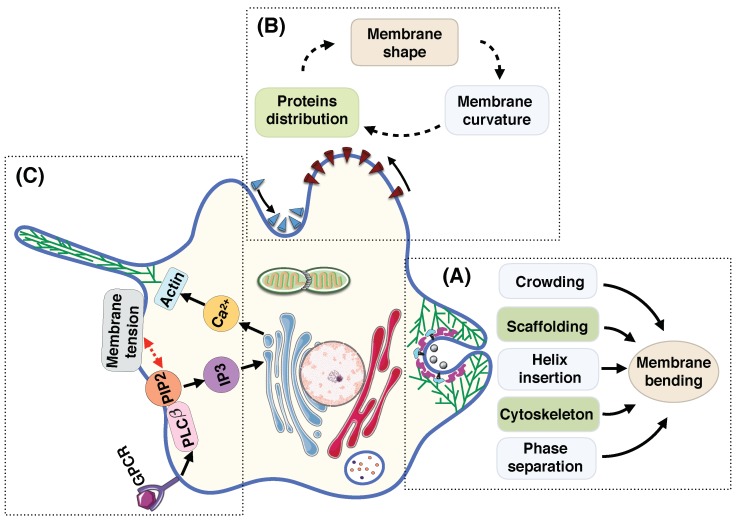
Perspective for the future of theoretical models for membrane curvature generating mechanisms. (**A**) Various mechanisms are involved in trafficking including amphipathic helix insertion into the bilayer, protein scaffolding, cargo-receptor crowding, forces from actin polymerization, and lipid phase separation [236,237]. (**B**) The coupling between membrane shape, membrane curvature, and membrane proteins distribution. The convex proteins (indicated with red cones) aggregate and flow toward the hill where the membrane curvature is negative (assuming the normal vector to the surface is outward). On the other hand, the concave proteins (represented by blue cones) accumulate and move toward the valley where the membrane curvature is large and positive [176]. (**C**) The coupling between the formation of a filopodial protrusion and the intracellular signaling inside the cell [238]. The ligand attachment to the G-protein-coupled receptor (GPCR) activates isotype β of the phospholipase C (PLCβ) which is a class of membrane-associated enzymes. PLCβ stimulates the phosphatidylinositol 4,5–bisphosphate (PIP2) which is a phospholipid component of the cell membrane and regulates the membrane tension. The hydrolysis of PIP2 produces the messenger molecule inositol trisphosphate (IP3). Binding IP3 molecules to the ER releases the calcium (Ca${}^{2+}$) that stored in the ER to the cytoplasm. The Ca${}^{2+}$ is a key intracellular molecule that controls the actin polymerization at the leading edge of the membrane protrusion.

- Membrane deformation is a dynamic process; surrounding fluid flow, thermal fluctuation, and diffusion of proteins actively regulate the shape of the membrane at each instance [11,188,225,226,227,228,229]. Currently, the models for membranes at mechanical equilibrium are well-developed but the models for dynamic processes have not been as well-developed and the community must invest some effort in this aspect.
- In vivo, multiple mechanisms coupling membrane deformation and cytoskeletal remodeling are commonplace (Figure 5A). Therefore, the models should be extended to include the dynamic effects and the rearrangement of the actin cytoskeleton layer underneath of the membrane.
- Membrane deformation and protein absorption/rearrangement are often considered as two separate processes with little to no impact on each other. However, recent studies show that proteins can sense the membrane curvature (Figure 5B). Therefore, there is a feedback loop between the protein distribution and the membrane configuration. While some models have considered this feedback loop [176,230,231,232,233], we still need more quantitative agreements between theory and experiment.
- Cell shape can control signal transduction at the plasma membrane, while intracellular signaling changes the membrane tension [234] (Figure 5C). This coupling between the cell shape and the signaling network inside the cell should be further understood in terms of both quantitative experimental and theoretical biology.
- As discussed above, membrane deformation is a multiscale phenomena that results from the reorientation of lipids to large-scale change in the membrane curvature. This suggests the extension of available models toward multiscale models that could represent each biological process over multiple length scales [117,235].

Despite these challenges, with increasingly quantitative measurement techniques available experimentally, ease of access to high throughput computing systems, and interdisciplinary training of the next generation of scientist leaders, the future of theoretical modeling of biological membranes and cellular membrane processes is brighter than ever.
